# A Multidisciplinary Model to Guide Employment Outcomes Among People Living With Spinal Cord Injuries in South Africa: A Mixed Methods Study Protocol

**DOI:** 10.2196/resprot.5887

**Published:** 2016-12-06

**Authors:** Ntsikelelo Pefile, Joyce Mothabeng, Saloshni Naidoo

**Affiliations:** ^1^ School of Health Sciences Division of Physiotherapy University of KwaZulu-Natal Durban South Africa; ^2^ Department of Physiotherapy School of Health Sciences Univeristy of Pretoria Pretoria South Africa; ^3^ Department of Public Health Medicine School of Nursing and Public Health University of KwaZulu-Natal Durban South Africa

**Keywords:** employment, spinal cord injury, vocational rehabilitation

## Abstract

**Background:**

Spinal cord injury (SCI) often results in complete or partial loss of functioning of the upper and/or lower limbs, leading to the affected individual experiencing difficulties in performing activities of daily living. This results in reduced participation in social, religious, recreational, and economic activities (employment). The South Africa legal framework promotes the employment and assistance of people with disabilities. However, rehabilitation interventions focus mainly on impairments and activity limitations, with few attempts to prepare those with SCI to return to gainful employment. There is therefore a need for a well-coordinated, multidisciplinary rehabilitation initiative that will promote the employment of people living with spinal cord injuries (PLWSCI) in South Africa.

**Objective:**

This study aims to develop a multidisciplinary model to guide employment outcomes amongst PLWSCI in South Africa.

**Methods:**

This study will utilize explanatory mixed methods during 3 phases. The first phase will explore the current rehabilitation practices, and the second will establish the factors that influence employment outcomes among PLWSCI. A multidisciplinary team consisting of health care professionals, representatives from the departments of Labour, Education, Social Development, and Health, and nongovernment organizations representing PLWSCI will provide feedback for the model development of phase 3, along with results from the previous 2 phases, using a multistage Delphi technique.

**Results:**

It is estimated that the results of phases 1 and 2 will be completed 11 months after data collection commencement (November 2015). Phase 3 results will be finalized 4 months after phases 1 and 2.

**Conclusions:**

Developing a multidisciplinary model to guide the employment outcomes of PLWSCI will ensure a coordinated response to integrate them into a productive life and will assist them to achieve economic self-sufficiency, personal growth, social integration, life satisfaction, and an improved quality of life. This can be achieved by active inclusion of PLWSCI to ensure that their concerns and recommendations are addressed.

**ClinicalTrial:**

ClinicalTrials.gov NCT02582619; https://clinicaltrials.gov/ct2/show/NCT02582619 (Archived by WebCite at http://www.webcitation.org/6mBgcj6z7)

## Introduction

### Overview

A spinal cord injury (SCI) is a life-transforming condition of sudden onset that can have devastating consequences [1]. It often results in complete or partial loss of functioning of the upper and/or lower limbs, and the affected individuals have difficulties in performing activities of daily living, which reduces their participation in social, recreational, and economic activities. An important activity for adults is the participation in productive work, as most societies expect people to work, with employment being regarded as a key indicator of social integration [2]. Employment provides people living with spinal cord injuries (PLWSCI) with economic and intrinsic rewards and enables greater life satisfaction and an improved quality of life. Globally, it is estimated that the employment rate of PLWSCI, following rehabilitation, ranges between 21% to 67% depending on age, gender, level of education, race, marital status, cause of injury, neurological level and classification, health status, degree of functional independence, time since injury and internal locus of control, access (including transport), and accommodation [2]. There is a paucity of information on the unemployment rate of people with disabilities (especially PLWSCI) in South Africa, which is estimated to be 25.2% [3]. However, this is compounded by a lack of information on the factors influencing employment and the absence of a rehabilitation framework aimed at improving employment outcomes among PLWSCI in South Africa.

In South Africa, a legal framework exists that promotes the employment as well as assistance of people with disabilities in the workplace. The Constitution of the Republic of South Africa 108 (1996) [4] clearly stipulates that all South African citizens (including PLWSCI) are equally entitled to the rights, privileges, and benefits (including employment) of citizenship [5]. Furthermore, chapter 2 of the constitution states that no citizen may be unfairly discriminated against (directly or indirectly) on one or more grounds (including disability). In addition, the Promotion of Equality and Prevention of Unfair Discrimination Act 4 (2000) [6] and the Employment Equity Act 55 (1998) [7] were promulgated to prevent unfair discrimination of people with disabilities and to promote their employment. The Labour Relations Act 66 (1995) [8], Skills Development Act 97 (1998) [9], Public Service Act 103 (1994) [10], and Basic Conditions of Employment Act 11 (1997) [11] were also enacted to guide employers (in private and public sector) in employing people with disabilities (including PLWSCI). The Integrated National Disability Strategy [12] and the South African National Rehabilitation Policy (2000) identified vocational rehabilitation as one of the key components of providing services to those affected. Rehabilitating individuals with SCI is intended to maximize their physical functioning and gainful employment so as to integrate them into their communities [13-15] and is often achieved through coordinated efforts in a multidisciplinary setting that includes physiotherapy.

There are 24 private and government-funded rehabilitation facilities in South Africa, none of which offers comprehensive rehabilitation programs that are multisectorial and multidisciplinary and facilitates employment of PLWSCI. Rehabilitation interventions provided in such institutions are mainly medical, with limited attempts to prepare those with SCI to return to gainful employment [16]. There is therefore a need for a well-coordinated, multisectorial, multidisciplinary, and multifactorial rehabilitation intervention that will promote the employment of PLWSCI in South Africa. Data on vocational rehabilitation practices, employment status, and factors influencing employment outcomes among PLWSCI are therefore required in order to develop a model that will guide the employment outcomes for affected individuals. Consequently, the aim of this study is to develop a multidisciplinary model to guide employment among people living with SCI in South Africa.

The objectives are to

1. Systematically review the literature on international vocational rehabilitation interventions for PLWSCI

2. Identify gaps in vocational rehabilitation practices by retrospectively analyzing the medical files of SCI patients

3. Explore vocational rehabilitation practices used by rehabilitation professionals among people who sustain SCI in KwaZulu-Natal, South Africa

4. Explore employment and its influencing factors amongst PLWSCI in KwaZulu-Natal

5. Determine the barriers and facilitators of employment among PLWSCI in KwaZulu-Natal

6. Triangulate information from phase 1 and 2 of the study and create aspects and structure of the proposed model

7. Validate and refine the model through consensus among stakeholders

8. Disseminate the model to stakeholders and relevant industry players in South Africa.

### Theoretical Framework

The conceptualization and development of this study is based on a combination of social justice theory, the social model of disability, international classification of functioning, disability and health. These are the guiding lenses to identify the variables to be assessed and the manner of their assessment and analysis. Social justice is about declaring the protection of equal access to liberties, rights, and opportunities, as well as taking care of the least advantaged members of society (including individuals living with SCI) [17]. Social justice is concerned with human well-being, which is described with 6 dimensions (related to each other) being identified as health, personal security, reasoning, respect, attachment, and self-determination [18]. For the purpose of this study, health, reasoning, respect, attachment, and self-determination will be explored.

Health is expressed as crucial to sustaining human existence across the full life span. Health in this context is described as more than the absence of biological malfunctioning or impairment and includes functional ability such as mobility, sight or hearing, pain, sexual dysfunction, infertility, and occupation [18,19]. Poor health is positively associated with poor employment outcomes in PLWSCI, often due to secondary complications such as pressure sores, depression, spasms, pneumonia, and heterotrophic ossification [20]. These and other conditions will be explored in this study to determine their impact on employment outcomes among PLWSCI using the International Classification of Diseases and Function (ICF). The ICF is a framework that is used to holistically assess the impairments, activity limitations, and participation restrictions in an individual with a disease or injury [21-24]. It assists professionals to establish the interactions of these domains with the environment to develop appropriate rehabilitation interventions.

It is further argued that reasoning includes analytical ability, imagination, the ability to form beliefs based on evidence (or experience), the ability to reflect on what counts as relevant evidence for those beliefs, and the ability to weigh the probative value of each [18]. In this study, this dimension will be explored by investigating the perspectives of PLWSCI regarding the impact of rehabilitation on their preparedness to participate in employment activities once discharged and by describing the factors that influence their participation in employment activities. PLWSCI will form an integral part of the team developing the proposed model to improve their employment outcomes.

Respect is an essential element of human flourishing and is an important concern of justice, being linked to self-respect [18]. The authors perceive respect as involving treatment of others as dignified moral beings, deserving of equal moral concern, which requires an ability to see people as independent sources of moral worth and dignity and view them as appropriate objects of sympathetic identification. Throughout the 3 phases of this study, this dimension will be achieved through the active participation of PLWSCI, which will be guided by the social model of disability.

The social model of disability is a paradigm that redefines disability in terms of the disabling environment and repositions disabled people as citizens with rights [25]. This model contends that society fails to provide appropriate services for PLWSCI and that barriers in society have to be removed for people with disabilities to fully participate in life situations (especially employment) [25-28]. Furthermore, the model stipulates that the programs or approaches developed to remove those barriers should be developed in consultation with people living with disabilities.

Attachment is the fourth dimension of Powers and Faden’s social justice theory [18]. They argue that forming bonds of attachment is one of the most central dimensions of human well-being. These bonds include friendship, love, solidarity, or fellow-feeling with others within their home, work, and social communities. In this study, the impact of employment on these relationships will be explored for PLWSCI and will give clarity on how they perceive their social role in interacting with fellow coworkers.

The last dimension put forward by Powers and Faden is self-determination, which is argued to be the ability to make decisions about one’s life plan [18]. While many PLWSCI are unable to control their physical mobility and their interaction with their physical environment, this does not mean that they should be excluded from being involved in making decisions about their lives. Therefore, developing a model that will attempt to improve employment outcomes in PLWSCI needs their active participation, this being in line with the principles enshrined in the social model of disability.

## Methods

### Overview

**Figure 1:**
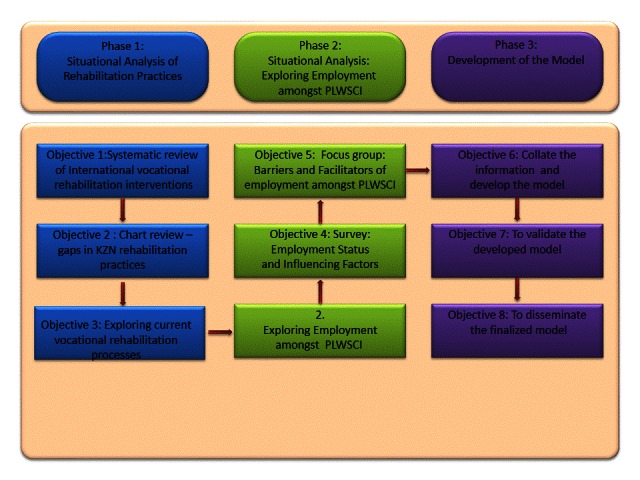
Methodology schematic diagram.

This explanatory mixed methods study will entail the collection and analysis of both quantitative and qualitative data [29-31]. An explanatory sequential approach will be used as described by Cresswell [30]. This study is divided into 3 phases, each of which consists of a number of stages to address the study objectives (see Figure 1).

Phase 1 of this study will address objectives 1 through 3 and will provide the background to current international and local practices regarding vocational rehabilitation strategies. This phase will also enable questionnaires and focus group schedules to be developed for discussion with stakeholders (rehabilitation professions, academics, nonprofit organizations, PLWSCI, insurers, and government representatives) for subsequent phases. Objective 1 will entail a systematic review of literature to identify the current best practices for vocational rehabilitation interventions. Objective 2 will identify gaps in vocational rehabilitation practices through retrospective review of the medical files of SCI patients at 2 regional spinal units in KwaZulu-Natal Province, South Africa [32]. Objective 3 will consist of semistructured interviews and focus groups with rehabilitation professionals and PLWSCI to establish the vocational rehabilitation services currently being rendered to SCI patients in KwaZulu-Natal Province.

Phase 2 will provide opportunities to engage with relevant stakeholders (PLWSCI, rehabilitation professionals, medical insures, and representatives from the KwaZulu Natal provincial departments of Education, Health, Social Development, Transport, and Labour) to determine the employment rate and associated factors of affected persons in postrehabilitation. Objective 4 will consist of structured interviews with PLWSCI to establish the contributing factors to their employment status since their injury. Objective 5 will explore the barriers and facilitators of employment among PLWSCI focus groups. The inclusion of several stakeholders will enable a range of perspectives to be explored, this being important for developing the model.

The results obtained from phases 1 and 2 will be used to develop and validate a model in phase 3. Objective 6 will entail triangulating information from phases 1 and 2 to create the aspects and processes of the proposed model. Objective 7 will consist of a focus group with the stakeholders who participated in phase 2 to validate the content of the proposed model and will entail obtaining consensus (after 3 rounds) among the rehabilitation professionals and academics regarding the structure and the content of the proposed model and using the modified Delphi technique [33-35]. The model will then be made available to relevant organizations, institutions, and publications for dissemination (objective 8).

### Data Collection Tools

Data collection tools for phase 1 (objectives 2 and 3) were developed using the International Spinal Cord Injury Core Data Sets [36,37] and the ICF checklists for individuals who sustained SCI [21,38,39]. The tool is divided into 5 sections: sections A and B contain information relating to the demographic profile (gender, age, marital status, population group, etc) of individuals who sustains SCI. Sections C and D contain information related to the injury and type of rehabilitation received in an acute or subacute setting. The last section of the tool contains information regarding the outcomes of rehabilitation and circumstances related to the discharge process. The interview schedule to be used to collect data to realize objective 3 contains questions relating to current rehabilitation practices and perceived barriers and facilitators of employment among PLWSCI from the perspective of the health care team (directly involved in caring for individuals who sustain SCI in the study setting). During the quantitative stage of phase 2 (objective 4), a data collection tool developed by the researcher using the literature [40-44] will be used. This tool is divided into 12 sections: sections A and B contain information relating to demographic information and employment history; sections C and D contain information relating to the attitudes toward employment and environment; sections E and F contain information relating to medical complications and social support; section G, H, and I contain attendance and bowel and bladder management information, and sections K, L, M, and N contain information relating to the injury, functional abilities, and quality of life. The tool will be assessed for validity and reliability in the study setting. The interview guide to realize objective 5 will be developed using the information obtained in objective 4. This tool will also be validated. Data collections tools for phase 3 will be developed once the data of phases 1 and 2 are analyzed. Moreover, SurveyMonkey (SurveyMonkey.com LLC) will be used to develop the questionnaire and undertake each Delphi round [35]. A 4-point Likert scale will be used to avoid neutral responses: agree, strongly agree, disagree, and strongly disagree). Each questionnaire will also have open-ended questions for participants to support their quantitative choices.

### Ethical Considerations

The study protocol received full ethical clearance from the University of KwaZulu-Natal (UKZN) Biomedical Research Ethics Committee (BE499/14) and the KwaZulu-Natal Provincial Department of Health (KZ_2015RP38_59) and is registered at ClinicalTrials.gov [NCT02582619]. Informed consent will be sought from all participants before they participate in the study.

### Study Setting and Participants

Objectives 2 and 3 of phase 1 will take place at 2 public sector hospitals that provide acute care services and the only public sector spinal rehabilitation unit in KwaZulu-Natal; the participants will consist of patients receiving care in these facilities and rehabilitation and health care professionals. Phase 2 will entail working with relevant nonprofit organizations to be able to access PLWSCI postrehabilitation as well as other stakeholders such as representatives from the private and public insurers, representatives from the state departments (Education, Health, Social Development, Transport, and Labour), and academics (who have a special interest in vocational rehabilitation and SCI care) invited to participate due to their expertise or role in providing services to people with SCI. The focus group discussions for objectives 7 and 8 (phase 3) will take place at the Physiotherapy Department, UKZN, Westville Campus, Durban, and will consist of rehabilitation specialists and PLWSCI, who will be provided with transport to and from the venue.

Multistage sampling technique will be used throughout the various phases of the study. A random sampling technique will be used to select participants during the quantitative parts of the study and purposive sampling during the qualitative (interviews and focus groups) parts. The sample size was calculated using the formula, n=*Z*^2^(1−α/2) pq/d^2^ where *Z* (1−α/2)=1.96 at 95% confidence interval (p=proportion of individuals with SCI admitted, q=1−p, and d=absolute allowable error). For this study, we presumed that *P*=.5 yields the largest possible sample size (maximum variability) if better approximation is not known, q=0.5, and precision (d)=±5%. This yielded a required size of 384 participants for phase 1.

Based on the finite population correction for estimated number of individuals who sustain a spinal cord injury previously seen in each facility over the last 5 years, the overall required sample size can be reduced to 295 for phase 1 (objective 2). Proportional sampling of each facility will be done based on the annual numbers of patients seen in each facility. Within each facility, files will be randomly selected to achieve the expected number of participants in each facility. The same formula was used to calculate the sample size for the quantitative phase 2 (objective 4) and yielded 123 participants. During the qualitative stages of phases 1 and 2 purposive sampling (representation of all study identified stakeholders) will be used to select participants. In phase 3, participants will be purposively selected with a representation from each group of the stakeholders for both the focus group and the Delphi rounds. Therefore, 18 participants will form part of the focus group and 30 participants will be identified in the subsequent Delphi rounds. During the interviews and focus group discussions, participants will be recruited in person and telephonically, after which they will be sent an email with a formal invitation to participate in the study and will also be requested to provide informed consent.

### Data Analysis

Throughout the phases, the quantitative data will be captured and analyzed using SPSS version 23 (IBM Corp). For objective 1, exploratory data analysis will be performed using a leaf and box plot to determine the distribution of the data to select the appropriate statistical tests. For objectives 1, 2, and 4 measures of central tendency and frequency distributions will be used to describe continuous and categorical data respectively. Depending on the distribution of the data, parametric and nonparametric tests will be used for bivariate analysis. Correlation efficient tests (Pearson correlation coefficient or Spearman rank correlation efficient) will be used to determine the relationship between the functional scores and the level of injury, the level of injury versus the length of stay, etc. Multiple linear regression and multiple logistic regression tests will be used to establish a relationship between demographic information and level of injury with the functional scores. The accepted level of significance will be less than .05. Analysis of data for objective 7 will include central tendencies such as median, mean, and mode and percentage score for each statement calculated to determine the level of dispersion and agreement respectively. The level of dispersion will be calculated for each statement using the interquartile range and standard deviation. Consensus will be defined as 70% or more of the participants being in agreement with a statement (that is participants scoring 4 (agree) or 5 (strongly agree), mean rating of more or equal than 3.5, and a coefficient of variation of less or equal to 30%. Kendall coefficient of concordance (*W*) will be calculated to measure consensus across participants. In consequent rounds (3) consensus will be acceptance of ratings higher than previously determined number by at least 51% of the participants and the elimination of topics that are vigorously opposed.

Qualitative data from objective 3 and 5 and part of objective 7 will be transcribed. To ensure accuracy, the research team will separately transcribe the information and compare and discuss discrepancies to reach a consensus using grounded theory. The written information will be checked for validity by the research team. Data will be uploaded to Nvivo version 10 (QSR International). This will be followed by content analysis during which the data from the interviews will be analyzed according to the following distinct but interconnected stages: Familiarizing (through reading and rereading transcripts as well as through listening to tapes over and over), identifying a thematic framework, indexing, charting and mapping, and interpretating the data as described by Raibee [45].

Attention will also be given to the nonverbal communication of interviewees. This will be noted on the interview schedule during the interview, and where nonverbal means of communication is evident on recordings (eg, lengthy silences, laughter), it will be added to transcripts. Data will be presented according to identified themes, with narrative examples being used to highlight each. Verification of data will be done through member checking and will continue with interviews and discussions until data saturation has occurred [46].

## Results

Phase 1 results will be triangulated and used to develop a guideline to integrate vocational rehabilitation during acute care and inpatient rehabilitation in the 2 public facilities that service people who sustain spinal cord injuries in KwaZulu-Natal. This phase will take 8 months to complete. The results from phase 2 will assist in describing the employment status, factors that influence employment, and barriers and facilitators of employment among PLWSCI. This phase will last for 3 months. The results from phases 1 and 2 will be integrated and used to develop the aspects and process of vocational rehabilitation model to improve employment outcomes. Once developed, the model will be validated through Delphi rounds. The last phase will last for 4 to 6 months.

## Discussion

Despite the existence of a good legal and policy framework that promotes the employment of people living with disabilities in South Africa, there is a scarcity of literature addressing the employment outcomes in affected persons during rehabilitation. Vocational rehabilitation is a broad term that includes a variety of services to assist an individual following an illness or disability [43]. It is also a multidisciplinary rehabilitation strategy that aims at enabling a disabled person to secure, retain, and advance in suitable employment [47,48]. It is concerned with supporting efforts made by a person with a disability to return to and maintain employment and includes vocational guidance and training, placement, employment, and other related services [47]. Vocational rehabilitation has been proven to be effective in improving employment outcomes in PLWSCI in developed and some developing countries [43,49-52]. However, there are no studies that have been done in Africa and South Africa on PLWSCI. This study will therefore develop a multidisciplinary and multisectorial model that will facilitate and guide employment outcomes for PLWSCI. This model will be developed in collaboration with affected persons, rehabilitation professionals, public service, private sector, and the insurance industry. It will propose and advocate strategies to implement policy imperatives that promote the employment of people living with spinal cord injuries in South Africa in an endeavor to improve their quality of life and sense of self-worth as contributing members of society. Further studies will be proposed that will assess the cost effectiveness and general effectiveness of various vocational rehabilitation approaches amongst PLWSCI, as this is beyond the scope of this study.

## Abbreviations

ICF: International Classification of Disease and Function
PLWSCI: people living with spinal cord injury
SCI: spinal cord injury
UKZN: University of KwaZulu-Natal

## References

[1] Harvey L (2008). Management of Spinal Cord Injuries: A Guide for Physiotherapists.

[2] Lidal I, Huynh T, Biering-Sørensen F (2007). Return to work following spinal cord injury: a review. Disabil Rehabil.

[3] Statistics South Africa (2015). Quarterly Labour Force Survey: 4th Quarter 2015.

[4] Paddison S, Middleton F, Stokes M, Stack E (2011). Spinal cord injury. Physical Management of Neurological Conditions.

[5] Republic of South Africa (1996). Constitution of the Republic of South Africa 108.

[6] Holness W, Rule S (2014). Barriers to advocacy and litigation in the equality courts for persons with disabilities. Potchefstroom Electronic Law Journal.

[7] Republic of South Africa (1998). Employment Equity Act 55.

[8] Republic of South Africa (1995). Labour Relations Act 66.

[9] Republic of South Africa (1998). Skills Development Act 97.

[10] Republic of South Africa (1994). Public Service Act 103.

[11] Republic of South Africa (1997). Basic Conditions of Employment Act 75.

[12] Republic of South Africa Office of the Deputy President.

[13] Harvey L (2008). Management of Spinal Cord Injuries: a Guide for Physiotherapists.

[14] Krause JS (2003). Years to employment after spinal cord injury. Arch Phys Med Rehabil.

[15] Tomassen P, Post M, van Asbeck FW (2000). Return to work after spinal cord injury. Spinal Cord.

[16] Pefile N (2014). Exploring Work Related Spinal Cord Injuries in Gauteng. Masters Thesis.

[17] Robinson M (2010). Assessing criminal justice practice using social justice theory. Soc Justice Res.

[18] Powers M, Faden R (2006). Social Justice: the Moral Foundations of Public Health and Health Policy.

[19] Nilsson I, Townsend E (2010). Occupational justice: bridging theory and practice. Scand J Occup Ther.

[20] Escorpizo R, Miller WC, Trenaman LM, Smith EM https://www.scireproject.com/rehabilitation-evidence/work-and-employment.

[21] Jette AM (2006). Toward a common language for function, disability, and health. Phys Ther.

[22] Cieza A, Kirchberger I, Biering-Sørensen F, Baumberger M, Charlifue S, Post M, Campbell R, Kovindha A, Ring H, Sinnott A, Kostanjsek N, Stucki G (2010). ICF Core Sets for individuals with spinal cord injury in the long-term context. Spinal Cord.

[23] Kirchberger I, Cieza A, Biering-Sørensen F, Baumberger M, Charlifue S, Post M, Campbell R, Kovindha A, Ring H, Sinnott A, Kostanjsek N, Stucki G (2010). ICF Core Sets for individuals with spinal cord injury in the early post-acute context. Spinal Cord.

[24] Herrmann K, Kirchberger I, Stucki G, Cieza A (2011). The comprehensive ICF core sets for spinal cord injury from the perspective of physical therapists: a worldwide validation study using the Delphi technique. Spinal Cord.

[25] Dewsbury G, Clarke K, Randall D, Rouncefield M, Sommerville I (2004). The anti‐social model of disability. Disabil Soc.

[26] Shakespeare T, Watson N (2001). The social model of disability: an outdated ideology?. Res Soc Sci Disabil.

[27] Massie B (1993). The Social Model of Disability. Physiotherapy.

[28] Barnes C (2000). A working social model? Disability, work and disability politics in the 21st century. Critical Social Policy.

[29] Kroll T, Neri M, Miller K (2005). Using mixed methods in disability and rehabilitation research. Rehabilitation Nursing.

[30] Cresswell JW, Clark VPL (2007). Choosing a mixed methods design. Designing and Conducting Mixed Methods Research.

[31] Cresswell JW, Clark VLP (2011). Designing and Conducting Mixed Methods Research, Second Edition.

[32] Hess R (2004). Retrospective studies and chart reviews. Respir Care.

[33] Vazquez-Ramos R, Leahy M, Estrada Hernandez N (2007). The Delphi method in rehabilitation counseling research. Rehabil Couns Bull.

[34] Hsu C, Sandford B (2007). The Delphi Technique: Making Sense of Consesus. Practical Assessement, Reseacrch and Evaluation.

[35] Rankin A, Rushton A, Olver P, Moore A (2012). Chartered Society of Physiotherapy's identification of national research priorities for physiotherapy using a modified Delphi technique. Physiotherapy.

[36] New PW, Marshall R (2014). International Spinal Cord Injury data sets for non-traumatic spinal cord injury. Spinal Cord.

[37] DeVivo M, Biering-Sørensen F, Charlifue S, Noonan V, Post M, Stripling T, Wing P (2006). International Spinal Cord Injury Core Data Set. Spinal Cord.

[38] Cieza A, Kirchberger I, Biering-Sørensen F, Baumberger M, Charlifue S, Post M, Campbell R, Kovindha A, Ring H, Sinnott A, Kostanjesek N, Stucki G (2010). ICF Core Sets for individuals with spinal cord injury in the long-term context. Spinal Cord.

[39] Biering-Sørensen F, Scheuringer M, Baumberger M, Charlifue S, Post MW, Montero F, Kostanjsek N, Stucki G (2006). Developing core sets for persons with spinal cord injuries based on the International Classification of Functioning, Disability and Health as a way to specify functioning. Spinal Cord.

[40] Krause J, Terza J, Dismuke C (2010). Factors associated with labor force participation after spinal cord injury. J Vocat Rehabil.

[41] Krause V, Dismuke C (2010). Factors associated with labour force participation after spinal cord injury. J Vocat Rehabil.

[42] Ottomanelli L, Lind L (2009). Review of critical factors related to employment after spinal cord injury: implications for research and vocational services. J Spinal Cord Med.

[43] Ottomanelli L, Barnett S, Goetz L, Toscano R (2015). Vocational rehabilitation in spinal cord injury: what vocational service activities are associated with employment program outcome?. Top Spinal Cord Inj Rehabil.

[44] Krause J (1992). Life satisfaction after spinal cord injury: A descriptive study. Rehabil Psychol.

[45] Rabiee F (2004). Focus-group interview and data analysis. Proc Nutr Soc.

[46] Domholdt E (2005). Rehabilitation Research: Principles and Applications.

[47] Gobelet C, Luthi F, Al-Khodairy AT, Chamberlain MA (2007). Vocational rehabilitation: a multidisciplinary intervention. Disabil Rehabil.

[48] Coetzee Z, Goliath C, van der Westhuizen R, Van Niekerk L (2011). Re-conceptualising vocational rehabilitation services towards an inter-sectoral model. S Afr J Occupat Ther.

[49] Marini I, Lee G, Chan F, Chapin M, Romero M (2008). Vocational rehabilitation service patterns related to successful competitive employment outcomes of persons with spinal cord injury. J Vocat Rehabil.

[50] Hansen CH, Mahmud I, Bhuiyan AJ (2007). Vocational Reintergration of People With Spinal Cord Lesion in Bangladesh-An Observational Study Based on a Vocational Training Project at CRP. Asia Pac Disabil Rehabil J.

[51] Khan LT, Ng Louisa, Turner-Stokes Lynne (2009). Effectiveness of vocational rehabilitation intervention on the return to work and employment of persons with multiple sclerosis. Cochrane Database Syst Rev.

[52] Ottomanelli L, Cipher D (2009). Employment and vocational rehabilitation services use amongst veterans with spinal cord injury. J Vocat Rehabil.

