# Correction: A Multidisciplinary Model to Guide Employment Outcomes Among People Living With Spinal Cord Injuries in South Africa: A Mixed Methods Study Protocol

**DOI:** 10.2196/13026

**Published:** 2019-04-03

**Authors:** Ntsikelelo Pefile, Joyce Mothabeng, Saloshni Naidoo

**Affiliations:** 1 School of Health Sciences Division of Physiotherapy University of KwaZulu-Natal Durban South Africa; 2 Department of Physiotherapy School of Health Sciences University of Pretoria Pretoria South Africa; 3 Department of Public Health Medicine School of Nursing and Public Health University of KwaZulu-Natal Durban South Africa

The authors of “A Multidisciplinary Model to Guide Employment Outcomes Among People Living With Spinal Cord Injuries in South Africa: A Mixed Methods Study Protocol” (JMIR Res Protoc 2016;5(4):e238) made a small copyediting error wherein the word “exploratory” should have read “explanatory”.

In the Methods subsection of the Abstract, the sentence:

This study will utilize exploratory mixed methods during 3 phases.

Has been revised to read:

This study will utilize explanatory mixed methods during 3 phases.

Additionally, in the first paragraph of the main Methods section, the sentence:

This exploratory mixed methods study will entail the collection and analysis of both quantitative and qualitative data [29-31]. An exploratory sequential approach will be used as described by Cresswell [30]. This study is divided into 3 phases, each of which consists of a number of stages to address the study objectives (see Figure 1).

Has been revised to read:

This explanatory mixed methods study will entail the collection and analysis of both quantitative and qualitative data [29-31]. An explanatory sequential approach will be used as described by Cresswell [30]. This study is divided into 3 phases, each of which consists of a number of stages to address the study objectives (see Figure 1).

The correction will appear in the online version of the paper on the JMIR website on April 3, 2019, together with the publication of this correction notice. Because this was made after submission to PubMed, PubMed Central, and other full-text repositories, the corrected article also has been resubmitted to those repositories.

